# Integrating social work into oral health care: a collaborative approach to achieving health equity

**DOI:** 10.3389/fpubh.2026.1716812

**Published:** 2026-02-04

**Authors:** Melanie Morris, Rachel S. John, Jamie Burgess-Flowers, Lisa de Saxe Zerden

**Affiliations:** 1 School of Social Work, University of Washington, Seattle, WA, United States; 2 Rutgers School of Social Work, New Brunswick, NJ, United States; 3 Workman School of Dental Medicine, High Point University, High Point, NC, United States; 4 University of North Carolina School of Social Work, Chapel Hill, NC, United States

**Keywords:** dental education, integrated care, mixed methods research, oral health equity, social work

## Abstract

**Background and purpose:**

Social workers in dental education champion the inclusion of social drivers of health and advocate for the needs of vulnerable communities using anti-oppressive, psychosocial-cultural, and systems approaches. However, despite these significant contributions, social workers’ unique roles and impact in these settings remain underexplored. This study examines these professionals’ role in advancing health equity in dental education settings.

**Methods:**

An explanatory sequential mixed methods design was used to explore the role and integration of social work in oral health settings. Data were collected from the Social Work in Dentistry (SWID) group, a national peer network of social workers who work in oral health settings. In the study’s first phase, quantitative data were collected via survey methods (*N* = 11) on their role. In the second phase of the study, one-time semi-structured interviews (*N* = 6) were conducted to gain a deeper understanding of participants’ perceptions of their roles, and their contributions to health equity. Mixed methods integration occurred in developing the semi-structured interview guide from the survey results and the analysis phase.

**Results:**

Social workers have multifaceted roles in dental education. Their roles included direct clinical practice (81.8%), patient care coordination (81.8%), dental education (90.9%), and supervising social work practicums (81.8%). When describing how their role contributes to health equity, the main themes that emerged were: (1) Increased access to dental care and community resources for patients, (2) educating the future dental workforce to address social drivers of health, and (3) influencing a new dental culture of practice.

**Conclusions and implications:**

This study highlights the unique perspectives and skills that social workers bring to oral health. In oral health, social workers play a dynamic role, engaging in cross-system collaborations that enhance patient and student outcomes. The findings demonstrate that these professionals, in collaboration with dental providers, can play a significant role in promoting health equity, leading to improved care delivery and outcomes for communities. Furthermore, integrating social workers into oral health settings shapes a new generation of dental providers better equipped to address patients’ psychosocial needs and deliver collaborative, person-centered care.

## 1 Introduction

Oral health disparities have become an indicator of social and structural inequities experienced throughout a person’s life. Poor oral health reflects a higher burden of disease, disparate access to health care, material hardship, and other physical and social inequities (1). Those who are low-income, uninsured, members of a racialized group, immigrants, and/or live in rural areas are more likely to have poor oral health (2). Poor oral health can result in increased risk for long-term chronic conditions, lost workdays and reduced employability, preventable dental-related hospital visits, and decreased overall well-being of a person (3). Most oral health diseases are preventable and/or treatable; however, dental caries remains a common chronic condition among all people (4).

Dental school clinics, community health centers, community hospitals, public and parochial schools, public health departments, and other social service agencies have become part of what is known as “the dental safety net” (5, 6). Dental safety nets are public and voluntary sector dental clinics that offer dental services at reduced or no cost (5). These clinics tend to attract dentally underserved populations, which also tend to be those who have poor oral health and face other social stressors and barriers to care, such as transportation issues, mental and general health complexities, and often need access to other public benefits (7). While dental schools are mainly serving those who experience many biopsychosocial challenges, dental students report not feeling comfortable in addressing these barriers to care (8).

Addressing these biopsychosocial barriers requires professionals with training that complements dentistry. Social workers bring this expertise, having long been part of the healthcare workforce at the individual, community, and system levels (9). Social workers are being increasingly hired in various health settings, such as hospitals and ambulatory care settings, to screen and assess patients for behavioral health needs, provide mental health interventions, facilitate communication among the healthcare team, coordinate care, and address social barriers to care among individuals and families (10, 11). Social workers have specific training in psychosocial risk factors, behavioral health intervention, cultural responsiveness, and person-in-environment practice while promoting and advancing health equity for all populations. This makes them well-positioned to strengthen oral health care teams, reduce inequities, and advance patient-centered care (12). Despite these established roles in healthcare, the dental field has been slow to integrate social workers.

### 1.1 History of social work in dentistry

**Figure 1:**
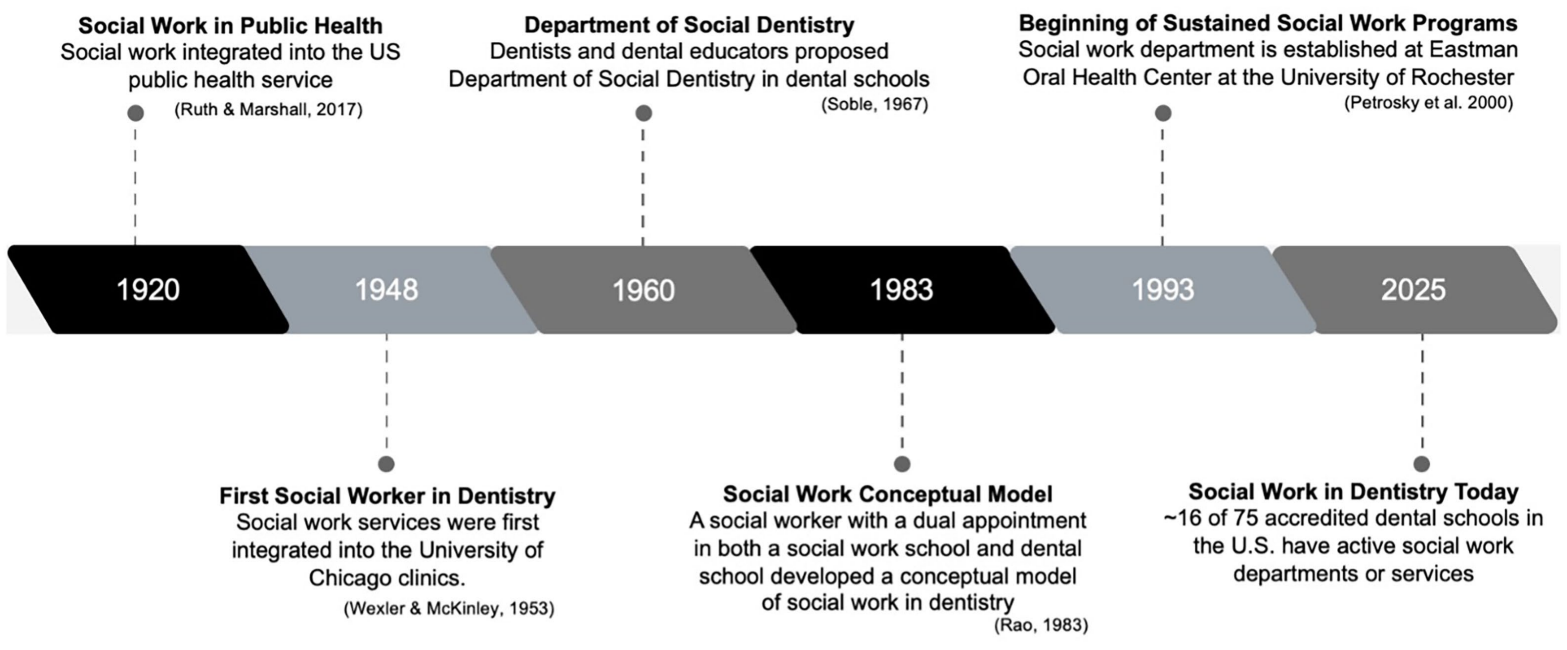
Timeline of social work integration in dental education.

Integrating social workers into the dental field, specifically dental education, is not new, as illustrated in Figure 1. The first dental social worker was hired at the University of Chicago Walter G. Zoller Memorial Dental Clinic in 1948 (13). The social worker’s focus was on addressing emotional and social barriers to manage caries risk and dental anxiety, however, the referrals fell into the following categories: (1) negative attitude toward or previous neglect of dental care, (2) difficulties in inter-family relationships, (3) social and environmental problems, and (4) serious medical or crippling conditions. In 1960, a dentist and dental educator proposed the establishment of a department of social dentistry in dental schools (14). They envisioned that this department would be responsible for developing a dental curriculum, teaching, and conducting research focused on social and behavioral aspects of patient care. Later in 1983, a social worker with a dual appointment in both a social work school and a dental school developed a conceptual model of social work in dentistry (15). This model expanded previous ideas of social dentistry by specifically naming the need for social workers to be in this role and including a direct service component of casework, counseling, advocacy, and interdisciplinary collaboration. Since then, social work and dental colleagues have collaborated to emphasize and expand social workers’ role as part of interprofessional teams in oral health settings by emphasizing the biopsychosocial aspects of health, social determinants, and their contextualization across the life course and among specific populations (e.g., pediatrics, special health care needs populations) (16–21).

There has been a growing movement to incorporate social workers into dental schools, championed by the growth of interdisciplinary collaboration and by the work of individual social workers and dental school faculty (22). This movement has translated into more dental schools hiring social workers as integral members of their clinics. However, in 2025, only 16 of the 75 (21.3%) accredited pre-doctoral dental schools have active social work services in their clinics. During the 2019–2020 academic year, social workers in these roles met and formed a peer network called Social Work in Dentistry (SWID). SWID brings together those who work in established social work departments, provide services within dental schools, or are advocating for new programs. The network meets monthly to exchange strategies for program development, discuss challenges, provide peer support, and identify opportunities to advance SWID’s mission of promoting oral health equity. This network has continued to grow new members through word-of-mouth and presentations at national conferences, such as the American Dental Education Association (ADEA).

### 1.2 Health equity approach

This study uses a health equity approach to explore how social workers contribute to dental education, recognizing that oral health is shaped by the multilevel influences between people, their communities, and structural factors. Health equity is defined as the “personal agency and fair access to resources and opportunities needed to achieve the best possible physical, emotional, and social well-being” (23). With this understanding of health equity, there is an emphasis on the various levels of influence needed to achieve it. The Health Equity Framework (HEF) (23), provides a conceptual model to highlight the explicit and implicit interactions of multilevel influences on health outcomes to influence practice. HEF identifies four interconnected spheres of influence that shift throughout the life course: (1) Systems of Power, (2) Relationships and Networks, (3) Individual Factors, and (4) Physiological Pathways. Systems of Power focuses on structural factors, such as policies, processes, and practices, that determine the distribution and access to resources and opportunities needed for health. Relationships and Networks focus on relational factors, such as connections with family, friends, community, school, and workplaces, that either promote healthy choices or enable health-harming behaviors. Individual Factors focus on a person’s response to social, economic, and environmental conditions through their attitudes, skills, and behaviors. Physiological Pathways focuses on an individual’s biological, physical, cognitive, and psychological abilities. HEF emphasizes that the spheres of influence evolve and shift over the course of someone’s life.

HEF was aimed as a tool for practitioner action and structural change, which aligns with the Grand Challenges of Social Work initiative. The social work profession adopted a grand challenge strategy to mobilize social work research, policy, practice, and education to address the most urgent social problems in the United States (24). One of the identified social problems was *Closing the Health Gap*, attributed to health inequities. Social workers are uniquely equipped to address health inequities because the profession is fundamentally oriented toward understanding and intervening in the social, cultural, political, and structural conditions that shape people’s lives. Social workers sit at the intersection of clinical care, community context, and structural systems and are uniquely trained to simultaneously address individual needs, institutional practices, and policy-level drivers of health. As health inequities persist amid socio-political division, shrinking public health investments, and growing barriers to care, social workers are essential to guiding interdisciplinary efforts that center communities, address structural factors, and move health systems toward equity (25).

Taken together, the HEF intertwined with social work training and professional values underscore that improving oral health equity requires attention to the individuals, relationships, and structural forces that shape health outcomes. This study uses these health equity approaches to interpret how social workers contribute to dental education in ways that respond to the multilevel influences impacting oral health. By grounding this study in health equity, it demonstrates how social workers help prepare an interprofessional workforce to recognize and address the social and structural drivers of oral health inequities.

### 1.3 The current study

The emergence and growth of SWID have underscored the promise of integrating social work into dental education and the critical gaps in understanding how these roles are established and sustained. Although dental schools serve large numbers of patients who face structural and social barriers to care and despite growing recognition of the value of interdisciplinary practice, little is known about how social workers experience their integration into dental settings, the barriers and facilitators that shape this process, and the ways their contributions advance oral health equity.

To address this gap, the present study builds on a prior study to explore the experiences and perspectives of social workers in dental education, focusing on their roles and perceptions of social work’s contributions to oral health through a health equity approach.

## 2 Methods and materials

### 2.1 Study design

This study used an explanatory sequential mixed methods design to address the specific research questions. An explanatory sequential design is a mixed-methods design in which a quantitative phase is followed by a qualitative phase to explain the initial results from the quantitative phase (26). From the original study, the quantitative phase revealed a multidimensional facet of the social work role. The qualitative phase was designed to examine these patterns in greater detail and situate them within a broader health equity framework. Since there is limited research that examines the role of social work in dental school settings, a mixed methods approach created a broader understanding of the role of social workers in this setting, provided a comprehensive analysis of the social workers’ experience, enhanced the validity of findings, and centered the voices of the practitioners (27, 28). This study was approved by the Institutional Review Board of the first author’s institution at the time of data collection (protocol number 6295X).

### 2.2 Data sources and collection

#### 2.2.1 Quantitative phase

During the quantitative phase, primary data collection aimed to identify general information on social work’s current role in oral health settings. A brief 10-to-15-min exploratory survey instrument was developed by three members of the research team, including two with social work practice experience in dental clinics (MM and JBF) and one researcher focused on social workers in integrated care (LDSZ). We piloted and tested all survey items to ensure content validity and revised them as needed.

The survey was administered through Qualtrics and asked participants about (a) demographic information, (b) history of social work program in the dental school, (c) social work’s current role in the dental school, (d) barriers and facilitators of social work integration in this setting, and (e) interest in participating in future research. Inclusion criteria for this phase of the study included: (1) an Adult aged 18 or older, (2) a social worker with at least a BSW, and (3) a formal full-time or part-time appointment in a school of dentistry. Comprehensive findings from this survey were reported in a prior publication (22). The present study used only survey data related to demographics, program information, and social work roles, which includes the question: *What is your role in the dental school? (Select all that apply): (1) Direct Clinical Practice with Patients, (2) Care Coordination with Patients, (3) Psychoeducation with Patients, (4) Patient Complaints, (5) Dental Student Education, (6) Research, (7) Other.*

Participants were recruited from the SWID network through email and monthly meeting announcements. At the time of study recruitment, only thirteen dental schools had social workers in their clinics, limiting the size of the eligible population. We received responses from twelve of these programs, a 92% response rate, reflecting substantial coverage of this emerging workforce.

#### 2.2.2 Qualitative phase

All participants who took the initial study survey were invited to a 30–60-min remote interview. An initial recruitment email outlined the study’s purpose and invited all eligible participants to join this qualitative phase. One follow-up email was sent. Participation was voluntary, and no incentives were provided. Of the twelve programs invited, six participated, resulting in a 50% response rate.

Building on the findings from the qualitative phase, a semi-structured interview guide was developed incorporating a health equity approach. This included the following topics: (1) history of the development of the social work role, (2) the role and perception of social work in the dental school, (3) social work’s role in health equity, and (4) barriers and facilitators of the role. The interview guide was piloted and tested with other members of the research team to ensure content validity and was revised as needed.

The first author (MM) coordinated and conducted all interviews. The interviews were recorded via Zoom with the participants’ consent. Audio recordings were transcribed using Rev. and reviewed for accuracy, de-identified, and corrected for minor errors.

### 2.3 Data analysis

#### 2.3.1 Quantitative analysis

All quantitative analyses used *Stata/SE version 19* (StataCorp, College Station, TX). Descriptive statistics were calculated to summarize sample characteristics and survey variables, including means, standard deviations, frequencies, and percentages. Missing data were examined. One program lacked sufficient data on key variables needed for this study and was therefore excluded from the full analytic sample. The final analytical sample for the quantitative strand was eleven.

#### 2.3.2 Qualitative analysis

A reflexive, inductive thematic analysis was conducted to explore the central research question from the qualitative interview transcripts using NVivo 14. The central research question for this phase was: *“What are social work’s contributions to oral health equity?”* Reflexive thematic analysis is a qualitative approach to analyzing qualitative data that establishes and highlights the researchers as central to the interpretation and meaning-making of the data (29). Two researchers (MM and RJ) independently reviewed the transcripts and developed the initial codebook. After coding the first transcript, the codebook was refined, and discrepancies were resolved through discussion. Both researchers coded all transcripts, and themes were developed iteratively through collaborative interpretation.

The researchers’ backgrounds and positionalities offered complementary insider and outsider perspectives that shaped the analytic process. One researcher was a social worker and oral health researcher who brought contextual knowledge of dental education, while the other was a social worker and health equity scholar focused on immigrant and refugee populations, contributing an external lens that challenged assumptions. The shared commitments to health equity and disciplinary training influenced what was prioritized in the data and the meanings that were emphasized. As part of our reflexive approach, we acknowledge that our interpretations were not neutral but situated and co-constructed with participants.

#### 2.3.3 Mixed methods integration

Quantitative and qualitative data were integrated in multiple ways throughout the study design. First, survey findings from the quantitative phase directly informed the development of the semi-structured interview guide in the qualitative phase, aiming to explore further the contextual, relational, and organizational factors shaping the survey findings. Second, during analysis, we compared results across phases to identify areas of convergence, expansion, or divergence, and interpreted these patterns through a health equity lens. Third, we developed a joint display to visually link quantitative constructs with qualitative themes, supporting a more integrated interpretation of the findings. Together, these strategies facilitated a coherent mixed-methods synthesis that extended the insights available from each phase alone (26, 30).

## 3 Results

Table 1 presents the demographics of the study sample (*N* = 11). The sample consisted of all women, with a mean age of 45.5 years (SD = 15.3). Most identified as White (72.7%), while 27.3% identified as Black or African American. Most held an MSW degree (81.8%), 18.2% held a PhD, and nearly all (90.9%) were licensed social workers in their state. Job titles included Social Worker (54.5%), Program Director (27.3%), and Assistant Professor (18.2%). On average, participants had been in their role for 5.4 years (SD = 4.7, range = 1–15). Regionally, participants were distributed across the Midwest (45.4%), Northeast (27.3%), and West (27.3%), with none from the South. The social workers represented 11 programs out of the 13 that participated in SWID at the time.

**Table 1:** Demographics of study sample (*N* = 11).

Demographic characteristic	Total sample (*N* = 11)
Age [m (SD)]	45.5 (15.3)
Female (%)	11 (100)
Race (%)	
Black or African American	3 (27.3)
White	8 (72.7)
Education (%)	
MSW	9 (81.8)
PhD	2 (18.2)
Licensed social worker (%)	10 (90.9)
Job title (%)	
Social worker	6 (54.5)
Assistant professor	2 (18.2)
Program director	3 (27.3)
Years in role [m (SD)]	5.36 (4.71)
Region of dental school (%)	
Northeast	3 (27.3)
Midwest	5 (45.4)
South	0 (0)
West	3 (27.3)

### 3.1 Role of social workers in dental education

**Figure 2:**
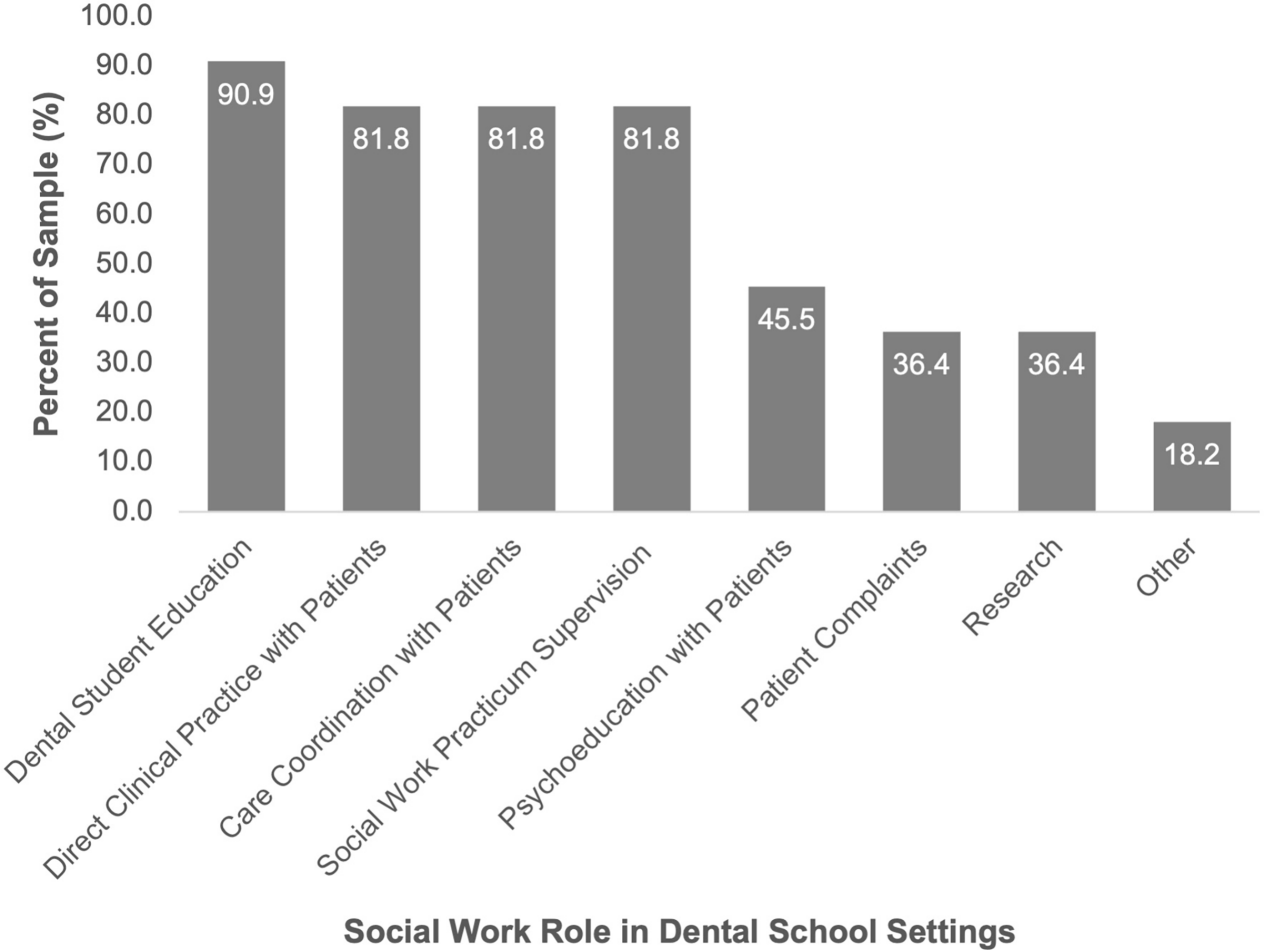
Social work roles in dental education.

Participants reported their professional roles across both the quantitative and qualitative phases. Figure 2 illustrates the range of roles identified in the survey. Nearly all participants (90.9%) reported responsibility in dental student education. Other commonly reported roles included direct clinical practice with patients (81.8%), patient care coordination (81.8%), and supervision of social work practicums (81.8%). Additional roles selected by participants included psychoeducation with patients, addressing patient complaints, research, advocacy, and clinic operations.

In the qualitative phase, all participants described their roles as multifaceted, which aligns with the quantitative data. Only one participant (9.1%) reported holding a single role in dental education. The remaining participants described multiple responsibilities, with some balancing patient care and education for dental and social work students (36.4%), while others reported these alongside additional responsibilities such as research, advocacy, and clinic operations (54.5%). One aspect of the role emphasized in the qualitative phase was that the individual social workers were tasked with developing and building their social work presence and services at each dental school, including designing social work practicum sites. As described by one participant:

“What’s unique about this position, at least for me, is that I was able to create it from scratch, which I really enjoy. And that’s cool because a lot of dental schools are not sure how to build out a program, what that means, what the components are, and what social workers should be doing. Should they just be doing clinical work? Should they just deal with the psychosocial stuff? I think it’s important to note that social workers are creative and versatile, so allowing them to build it out can be really beneficial in creating a strong, comprehensive program.” (P5)

However, because each program developed its own services and responsibilities, the social work role has often shifted and lacked consistency in day-to-day activities across dental schools. For example, in direct clinical practice, some social workers may focus on providing assessments for various needs (e.g., smoking cessation, dental anxiety), others are mitigating various barriers that may arise to patients completing care (e.g., insurance coverage, transportation), while others are addressing acute psychosocial and safety needs (e.g., domestic violence, legal guardianship for minors). This also applies to direct and clinical education responsibilities. Some social workers are developing courses for the behavioral science accreditation requirements, while others are developing courses and lectures on interprofessional practice, communication, and social drivers of health. One participant described this as a challenge, particularly when comparing how programs were used within their institutions:

“Every college of dentistry defined [social work] differently. It varies from school to school, what your title is, whether you are faculty, and what expectations are tied to the job. Not having a clearly defined position across colleges made it harder because everyone could put their own spin on it. That flexibility can be helpful, as schools can say, ‘we need you in pediatrics’ or ‘we need you in the whole college,’ but it also contributes to a lack of clarity.” (P4)

These findings highlight that social workers in dental education take on multifaceted, creative, and adaptive roles that are not consistently defined across schools. While there is overlap in the general responsibilities, participants highlighted that the day-to-day activities looked very different from program to program. This variation can create ambiguity, but it also shows the versatility of the profession and the ability to meet a range of needs for patients, students, and faculty.

### 3.2 Social work’s contribution to oral health equity

From the comprehensive quantitative findings, a key aspect further explored in the qualitative interviews was how social workers play a central role in advancing equity within dental education and practice. Three themes emerged, highlighting how social workers’ multifaceted roles contribute to equity across multiple, interrelated levels of influence. Table 2 summarizes each theme and provides exemplar quotes.

**Table 2:** Overview of themes that illustrate social work’s contribution to oral health equity.

Themes	Definition	Example quotes
Increased access to dental care and community resources for patients	This theme captures how social workers expand pathways to care by addressing barriers that limit patients’ ability to seek and complete treatment. Through case management, navigation, and referrals, social workers connect patients to affordable dental services and broader community resources such as housing, food, transportation, or language interpretation.	“Had I not been made aware of that and had I not had an opportunity to influence that, that patient would’ve been dismissed. When you have a mission statement of serving the underserved and whole person health, there is an obligation to go the extra mile or two to find out what’s happening with people. Dental providers and administrators do not always have the time to do that, and I do not always have the time to do it either, but I think whoever has the time to do it, it needs to be done. Because, people deserve to be treated with that level of dignity and respect.” (P1) “We are able to see the patient in a way that other people cannot. And so while yes, everyone is there for dental treatment, there are a lot of other needs that our patients have. And so using social work enables us to address some of those needs that would better enable someone to focus on their oral healthcare, right? By using the social worker, we can kind of level the playing field so that people can focus on this aspect of their health.” (P2)
Educating the future dental workforce to address social drivers of health	This theme highlights the role of social workers in shaping dental students’ understanding of the broader social and structural factors that influence oral health. Through didactic teaching, clinical mentoring, and interprofessional collaboration, social workers expose dental students to concepts such as health equity, cultural humility, trauma-informed care, and systemic barriers to care.	“I noticed that we had a gap in talking about working with patients who were transgender, which it got expanded to a lecture about working with patients from the LGBT community. And what are the things that we need to think about, which then became an in-service presentation to faculty as well. And so getting them to think about the whole picture. If we are going to talk about equity and diversity, that’s great, but how do we actually do that in a practical, tangible way? And so I love teaching. I love that role and getting to see kind of that light bulb moment where it clicks.” (P2) “I think a big piece that we do provide is the ability to provide the tools and the resources to our students here and our residents so that they can continue care, complete care for these residents. And I think we are also, in a way, I would not say changing, but we are enhancing or we are making the students, residents and faculty think differently, changing their perspective a bit on how they view underserved populations and looking at health disparities and health equities and looking at it just from a different lens that allows a little bit more of an empathetic approach to the care that they do versus thinking in a very close ended lens, which is just dentistry and getting it done.” (P5)
Influencing a broader culture of dental practice	This theme emphasizes how social workers contribute to shifting the norms, values, and practices of dental education and clinical environments. By introducing patient-centered, equity-focused, and trauma-informed approaches, social workers help foster a culture that prioritizes compassion, collaboration, and responsiveness to patients’ social contexts. Over time, this influence reshapes institutional practices and professional identities, promoting a dental culture that recognizes and addresses social drivers of oral health inequities.	“Interviewee, you know, you are coming in and talking with people who literally have been trained to have a two millimeter view.” “And they were saying how the glasses that they use, like zoom in and make… So I want to check that actually two millimeters, but it’s like it zooms into like two millimeters, right? And that’s their focus it’s on that cavity or on that whatever. And that’s their view. And you are asking them to go back to a like thousand foot view of a person. And so it’s a huge culture shift, way more than I think any primary care provider has needed to make.” (P0) “I think the students they are so used to that small view of looking at, and they are working very small, small part of a small tooth, whatever, but thinking about how are the things happening around people and their relationships and their social support and their living environment, the social determinants of health, how does that affect them? And they are therefore their dental care whether directly or indirectly and shutting light on some of those things and how they can be barriers or enablers to dental care so that our students are thinking more about person-centered care and thinking about the whole person beyond just their oral healthcare needs.” (P3)

#### 3.2.1 Theme 1. Increased access to dental care and community resources for patients

The first theme that emerged was *increased access to dental care and community resources for patients*. Participants consistently emphasized that social workers expand pathways to dental care by addressing barriers that often prevent patients from seeking or completing treatment. Through case management, navigation, and referrals, social workers connected patients to affordable dental services and broader community resources such as housing, food, transportation, and interpretation services. As one participant explained:

“We increase or improve access to dental care, [removing] those barriers that can either hinder or even prevent altogether dental care from happening like those financial barriers or distance or mobility.” (P3)

Another participant described the challenge of patients assuming dental schools are affordable only to discover care remains out of reach:

“People recognize we’re losing a whole population just because of finances. They come in thinking the dental school’s going to be affordable, and they realize it’s still not affordable, it’s still a lot of money.” (P0)

For some, social workers’ approach to care and advocacy ensured that patients were not dismissed prematurely. One participant discussed how one of their long-time patients, who had a consistent history of successful treatment, suddenly stopped attending their appointments. The clinic was going to dismiss the patient from the clinic, discontinuing their dental care and access to the clinic, but the social worker took it upon herself to explore why there was a sudden change. The social worker discovered through conversations with the patient that they had recently had a child with complex medical conditions, and there were no reliable childcare supports for them to attend their appointments. The participant describes the importance of her role:

“Had I not been made aware of that and had I not had an opportunity to intervene, that patient would’ve been dismissed. When you have a mission statement of serving the underserved and whole-person health, there is an obligation to go the extra mile…people deserve to be treated with that level of dignity and respect.” (P1)

Participants also highlighted how social workers addressed broader systemic barriers. One social worker described helping a patient who was distrustful of healthcare access due to past medical treatments:

“We are able to see the patient in a way that other people can't. And so while yes, everyone is there for dental treatment, there are a lot of other needs that our patients have. And so using social work enables us to address some of those needs that would better enable someone to focus on their oral healthcare, right? I got a referral for a patient whose blood pressure was high and they needed to go to the doctor, but they were refusing to go to the doctor because they had unnecessary treatment previously. They were really untrusting of the healthcare system. And so certainly by making sure that this patient has access to a doctor who looks like them, who can explain things thoroughly, puts them in a better head space, gives them better care. But then we can focus on their dental needs because as a result of their blood pressure being high, we couldn't give them any treatment. And so these barriers physically prevent people from getting treatment. And so by using the social worker, we can level the playing field so that people can focus on this aspect of their health.” (P2)

Through these efforts, social workers filled critical gaps in access, ensuring patients who might otherwise “fall through the cracks” were connected to care (P4). Collectively, these contributions demonstrated how social workers advanced equity by helping patients overcome social and structural barriers to oral healthcare.

#### 3.2.2 Theme 2. Educating the future dental workforce to address social drivers of health

In addition to patient-facing work, participants underscored social workers’ unique role in preparing dental students to recognize and respond to social and structural influences on oral health. Through didactic teaching, clinical mentoring, and interprofessional collaboration, social workers integrated concepts such as health equity, cultural humility, trauma-informed care, and systemic barriers to treatment into dental training.

One participant described the excitement of watching students begin to think beyond individual patient encounters:

“This other group [of dental students] hasn’t quite started working with patients, and they want to be the best providers possible. They’re actually thinking not just about patient care, but about shifting their workflow, shifting their practice… systemic changes they could make. That’s probably been the most rewarding piece, seeing them think about big changes, not just the little c changes, but the big C Changes.” (P0)

Another highlighted how experiential assignments shaped student learning and collaborating with social workers to address patients’ whole health:

“I introduced an assignment, an interprofessional assignment where the D3 students have to make a social work referral. In the assignment, they make the referral, follow up, and then write a reflection about the experience.” (P2)

Participants also described efforts to expand curricula to better prepare students to serve diverse populations, such as developing training on working with transgender patients or the broader LGBTQ+ community:

“If we’re going to talk about equity and diversity, that’s great, but how do we actually do that in a practical, tangible way? That’s what I love—getting them to think about the whole picture and seeing that light bulb moment where it clicks.” (P2)

These contributions expanded dental students’ knowledge base and gave them practical, equity-oriented tools for patient care. As one participant summarized:

“We’re making the students, residents, and faculty think differently, about underserved populations, about health disparities, about equity. They’re learning to take a more empathetic approach rather than thinking in a very close-ended lens of just dentistry and getting it done.” (P5)

#### 3.2.3 Theme 3. Influencing a broader culture of dental practice

Finally, participants highlighted how social workers helped shift the culture of dental practice toward a more equity-focused model of care. By introducing patient-centered, trauma-informed, and interprofessional approaches, social workers encouraged dental faculty, staff, and students to broaden their perspective. One participant described this shift as helping providers move beyond a narrow clinical focus:

“Dentists are trained to have a two-millimeter view, it zooms in on that cavity or that tooth. And you’re asking them to go back to a thousand-foot view of a person. It’s a huge culture shift.” (P0)

Others emphasized that the presence of social workers reassured students, faculty and staff that they were not alone in managing complex cases. While dentists often work intraprofessionally across dental specialties, patients frequently present with medical and social complexities outside providers’ comfort zones to address. In these situations, interprofessional collaboration was essential for supporting the patient and easing the burden on providers. As one participant explained,

“Something less tangible is just the idea that you’re not alone in this. You don’t have to face a difficult situation with a student or a patient by yourself. You have someone to come alongside you and help shoulder the load.” (P1)

Over time, this influence began to reshape institutional identity and practice. As one participant reflected:

“When I came on board, I didn’t want to be a band-aid approach or a one-time intervention. I wanted to develop systems and best practices and bring in the social work model of care. Over time, we became more of the fabric of the institution.” (P5)

Through these shifts, social workers contributed to building a dental culture that prioritizes compassion, collaboration, and responsiveness to patients’ broader social contexts, directly aligning dental education with equity goals.

### 3.3 Integrated findings

**Figure 3:**
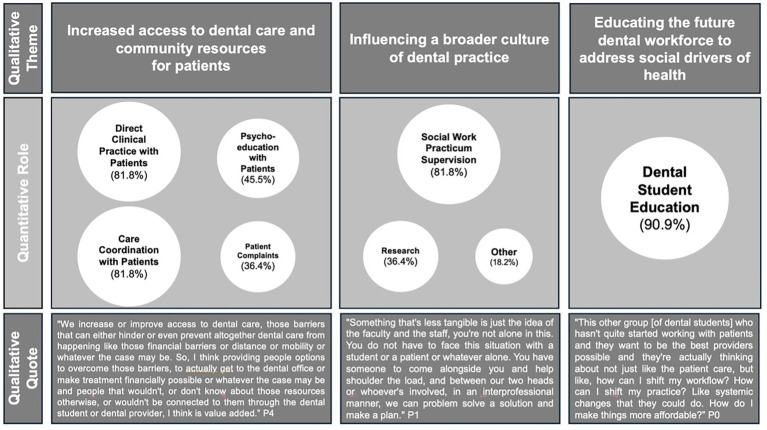
Joint display of quantitative roles and qualitative themes highlighting how social workers contribute to equity in dental education.

The joint display (Figure 3) illustrates how participants’ reported roles are embedded with their reflections on how those roles advance equity across patient care, student learning, and institutional culture. The quantitative survey identified the roles of social workers across various dental schools. These findings highlighted the multifaceted roles they play and how these roles interface with patients, students, faculty, and institutional practices. The qualitative themes reinforced and provided context for the survey results. In practice, social workers addressed barriers that prevent patients from accessing or completing treatment, such as financial strain, transportation, or distrust of healthcare, through direct clinical care (81.8%), care coordination (81.8%), psychoeducation (45.5%), and resource referrals. These activities ensured that vulnerable patients were not left behind, reflecting a commitment to dignity and whole-person care. At the same time, social workers prepared the future dental workforce to understand and respond to social drivers of health, with nearly all participants engaged in dental student education (90.9%). Through teaching and mentoring, they helped students think beyond technical procedures to consider affordability, cultural humility, and systemic change. Finally, by supervising practicums (81.8%), leading research (36.4%), and embedding interprofessional collaboration, social workers contributed to shifting the culture of dental schools toward more equity-focused and person-centered practices.

Together, the integration of these findings demonstrates that social work in dentistry is not an ancillary service but a multilevel mechanism to support a more just and inclusive oral health system. Considering the HEF, the social work roles and impacts in dental education engage all four of the spheres of influence. For example, social workers’ direct clinical care, care coordination, and psychoeducation help patients overcome barriers to accessing and utilizing dental services (Theme 1). These activities support Individual Factors and Physiological Pathways, build the Relationships and Networks necessary for equitable care, and contribute to more equity-focused policies and practices within Systems of Power. This also reinforces that social workers operate at the intersection of multiple influences, and therefore uniquely positioned to drive equity-oriented transformation across clinical care, workforce development, institutional culture, and ultimately expand how oral health is understood more broadly.

## 4 Discussion

This study is among the first to empirically explore the role of social workers across various dental schools and to examine how these roles contribute to advancing equity in oral health. Findings demonstrate that while social workers take on diverse and multifaceted responsibilities, their contributions converge across three interrelated pathways: increasing access to care for patients, educating future dental providers about social drivers of health, and shaping a broader culture of practice within dentistry. Collectively, these findings highlight the centrality of social work to advancing equity in oral health education and practice.

Participants described social workers as playing a critical role in reducing barriers to care and connecting patients to needed community resources. This aligns with prior literature showing that unmet social needs, such as transportation, insurance, and language barriers, contribute significantly to disparities in oral health access and outcomes (2, 31–34). By addressing these barriers, social workers expanded access and completion of treatment and ensured that vulnerable patients were not excluded from care.

Findings also show that social workers contribute to the training of dental students and residents by embedding health equity into curricula, mentoring, and experiential learning opportunities. This aligns with national calls to integrate social determinants of health into health professions education (35–37). Social workers’ ability to guide students in understanding systemic barriers, practicing cultural humility, and engaging in trauma-informed care provides future dentists with skills critical for delivering patient-centered and equitable oral healthcare. The emphasis on preparing students to think beyond technical care toward “*big C Changes*” as one participant explained, underscores social work’s role in shaping a workforce capable of addressing inequities at multiple levels.

Beyond individual patients and students, participants described social workers as influencing the broader culture of dental schools. By reframing how dental faculty and students approach patient care, social workers helped institutions shift from a narrow clinical lens toward a more holistic, person-centered perspective. This cultural shift aligns with broader movements in healthcare toward interprofessional collaboration and team-based care (12, 38, 39). Importantly, social workers not only supported patients but also reassured dental providers, reducing feelings of isolation and discomfort when managing complex cases. Over time, their contributions became embedded in institutional practices, underscoring that social work is not a supplemental service but an integral part of dental education.

Importantly, participants emphasized that these roles were not standardized across schools, suggesting both a challenge and an opportunity. The lack of role definition can result in unclear expectations, inconsistent integration within clinical teams, and limited institutional investment. This inconsistency may also present challenges to the development of shared metrics to evaluate social work’s impact on patient care, student learning, and clinic operations. However, participants also described this variability as a strength. This enables social workers to shape their roles around the specific social, structural, and cultural needs of their patient populations, and to respond quickly as new issues emerge. As dental schools increasingly confront inequities in access, treatment completion, and trust, the adaptability of social work offers a promising mechanism for advancing equity-focused care.

These findings suggest several implications for dental education and oral health equity. First, establishing clearer role definitions and sustainable structures for social work within dental schools may help address the current variability while maintaining flexibility to meet local needs. Second, integrating social workers into accreditation standards related to behavioral sciences, interprofessional education, and community engagement may strengthen the equity orientation of dental training. CODA standards emphasize person-centered care, interprofessional collaboration, and attention to social determinants of health. Explicitly integrating social work into CODA’s expectations would not only clarify role definitions but would also signal that equity-oriented care is foundational to dental education rather than optional. Finally, as dental schools increasingly confront the realities of structural inequities in oral health, social workers are uniquely positioned to help bridge clinical care with community-based solutions. While this study focused specifically on dental education settings, there is a significant opportunity to explore the role of social work iin Federally Qualified Health Centers (FQHCs) that provide dental services and other safety-net dental practice environments, where similar equity challenges exist. Findings from this study, and others, strengthens the evidence to show how the social work profession can positively contribute to patient care, workforce development, and systemic change.

Notably, despite the study’s findings, it has several limitations. The small, non-random sample reflects social workers only in the SWID national network, which may limit generalizability. The sample also consisted entirely of women and lacked representation from the U.S. South, where structural and policy barriers to oral health are distinct. At the time of data collection, we were only aware of 13 U.S. dental schools that employed social workers, and many programs do not publicly identify these roles, making it challenging to recruit this population. Although the final analytic sample was small (Quantitative Strand = 11, Quantitative Strand = 6), the response rate among these known programs was high (Quantitative Strand = 92%, Qualitative Strand = 50%), suggesting strong representation of the current field. To protect confidentiality and participant identification of the small sample, we did not name the institutions that participated in the study, only reported the regional locations, and carefully reviewed qualitative excerpts to ensure they did not contain identifiable details.

These limitations reflect the nascent nature of this workforce and may impact generalizability. Nonetheless, documenting the role of social workers in dental education is relatively new. There has been gradual growth, and since the formation of SWID in 2019, only a few additional programs have emerged. Future research should examine how roles are sustained and funded across diverse institutional contexts, and how the presence of social workers influences patient outcomes, student learning, and institutional policies over time. This includes assessing the economic value of these positions, examining sustainability beyond grant funding, and comparing models across schools and safety-net clinics to clarify how programs maintain these positions. Researchers should also explore recruitment strategies to identify social workers beyond SWID and capture the experiences of programs in regions and institutional types (e.g., FQHCs, Community Health Clinics, Hospital Dentistry) not represented in this study.

Overall, social workers in dental education have multifaceted and evolving roles central to advancing oral health equity. By bridging patient access, educating future dental providers, and influencing institutional culture, social workers help create more just and inclusive pathways to care. These findings suggest that rather than being peripheral, social work is integral to the mission of dental education and should be recognized as a vital contributor to oral health equity.

## Data Availability

The raw data supporting the conclusions of this article will be made available by the authors, without undue reservation.
