# Supplementary material for: Integrating social work into oral health care: a collaborative approach to achieving health equity
Source: Front Public Health. 2026 Feb 4;14:1716812. doi: 10.3389/fpubh.2026.1716812 (PMC12913468; doi:10.3389/fpubh.2026.1716812)
Supplement: Supplementary file 1 [file Data_Sheet_1.pdf]

8 Appendix

8.1 Survey

Start of Block: Demographic Questions

Q1 How do you currently describe your gender identity?

- ○ Male (1)
- ○ Female (2)
- ○ Non-binary / third gender (3)
- ○ Other: (4) ____
- ○ Prefer not to answer (5)

Q2 What is your age in years?

- ○ Please Specify: (1) ____
- ○ Prefer not to answer (2)

Q3 Which categories best describe you? (Select all that apply to you)

- ☐ American Indian or Alaska Native—For example, Navajo Nation, Blackfeet Tribe, Mayan, Aztec, Native Village of Barrow Inupiat Traditional Government, Nome Eskimo Community (1)
- ☐ Asian—For example, Chinese, Filipino, Asian Indian, Vietnamese, Korean, Japanese (2)
- ☐ Black or African American—For example, Jamaican, Haitian, Nigerian, Ethiopian, Somalian (3)
- ☐ Hispanic, Latino or Spanish Origin—For example, Mexican or Mexican American, Puerto Rican,Cuban, Salvadoran, Dominican, Colombian (4)
- ☐ Middle Eastern or North African—For example, Lebanese, Iranian, Egyptian, Syrian, Moroccan, Algerian (5)
- ☐ Native Hawaiian or Other Pacific Islander—For example, Native Hawaiian, Samoan, Chamorro, Tongan, Fijian, Marshallese (6)
- ☐ White—For example, German, Irish, English, Italian, Polish, French (7)
- ☐ Some other race, ethnicity, or origin, please specify: (8) ____
- ☐ Multi-racial, please specify: (9) ____
- ☐ Prefer not to answer (10)

Q4 What is your highest level of education?

- ○ Bachelor’s degree (1)
- ○ Master’s degree (2)
- ○ Doctorate degree (3)
- ○ Other: (4) ____
- ○ Prefer not to answer (5)

Q5 Do you have a social work degree?

- ▢ Yes - Bachelor's Degree (BSW) (1)
- ▢ Yes - Master's Degree (MSW) (2)
- ▢ Yes - Doctorate Degree in Social Work (4)
- ▢ No - My Degree is in: (3) ____

Q6 Are you a licensed social worker in the United States? (For example: LMSW, LCSW, etc.)

- ○ No (1)
- ○ Yes, please specify: (2) ____

Display This Question:

If Are you a licensed social worker in the United States? (For example: LMSW, LCSW, etc.) = Yes, please specify:

Q6a What state are you licensed in?

____

Q7 What is your current job title?

____

Q8 How many years have you been in your role?

____

Q9 Is your position defined as full time?

- No (1)
- Yes (2)

Display This Question:

If Is your position defined as full time? = No

Q9a How many hours a week do you work?

____

End of Block: Demographic Questions

Start of Block: Dental School Questions

DSQ. The following section will ask you questions related to your role within your dental school/institution.

Q10 What dental school do you work in?

____

Q11 Are you considered a faculty member within the dental school?

- ○ No (1)
- ○ Yes (2)

Display This Question:

If Are you considered a faculty member within the dental school? = No

Q11a Are you considered a staff member?

- ○ No (1)
- ○ Yes (2)

Q12 What is the name of your service/office in the dental school? (For example: PATH, CARES, etc.)

- ○ Name: (1) ____
- ○ No official name (2)

Q13 In what year was the social work service/office implemented in your dental school?

____

Q14 Not including yourself, how many other paid employees are part of the social work service/office within your dental school?

- ○ 0 (1)
- ○ 1 (2)
- ○ 2 (3)
- ○ 3 (4)
- ○ Other, specify number: (5) ____

Display This Question:

If Not including yourself, how many other paid employees are part of the social work service/office... = 1

And Not including yourself, how many other paid employees are part of the social work service/office... = 2

And Not including yourself, how many other paid employees are part of the social work service/office... = 3

And Not including yourself, how many other paid employees are part of the social work service/office... = Other, specify number:

Q14a Are all of the paid employees social workers?

- ○ No (1)
- ○ Yes (2)

Display This Question:

If Are all of the paid employees social workers? = No

Q14b What is the degree and/or title of the other paid employees who are not social workers?

____

Q15 What is your role in the dental school? (Select all that apply)

- [ ] Direct Clinical Practice with Patients (7)
- [ ] Care Coordination with Patients (1)
- [ ] Psychoeducation with Patients (2)
- [ ] Patient Complaints (3)
- [ ] Dental Student Education (4)
- [ ] Research (5)
- [ ] Other: (6) ____

Display This Question:

If What is your role in the dental school? (Select all that apply) = Dental Student Education

Q15a What courses do you teach in the dental school?

____

Q16 Which dental clinics/specialties do you work with? (Select all that apply)

- ☐ Pre-doctoral Student Clinic (1)
- ☐ Pediatric Clinic (2)
- ☐ Endodontic Clinic (3)
- ☐ Periodontic Clinic (4)
- ☐ Orthodontic Clinic (5)
- ☐ Prosthodontic Clinic (6)
- ☐ Oral and Maxillofacial Surgery Clinic (7)
- ☐ Advanced Education in General Dentistry (AEGD) Clinic (8)
- ☐ General Practice Residency (GPR) Clinic (9)

End of Block: Dental School Questions

Start of Block: Social Work Questions

SWQ. The following section will ask you questions about your affiliation to school/program of social work and your role as a social work intern supervisor.

Q17 Are you affiliated with a school/program of social work?

- ○ No (1)
- ○ Yes (2)

Display This Question:

If Are you affiliated with a school/program of social work? = Yes

Q17a What school/program of social work are you affiliated with?

____

Display This Question:

If Are you affiliated with a school/program of social work? = Yes

Q17b Are you considered faculty at this school/program of social work?

- No (1)
- Yes (2)

Display This Question:

If Are you affiliated with a school/program of social work? = Yes

Q17c What responsibilities do you have at the school/program of social work? (For example: Teach courses, supervise students, etc.)

____

Q18 Do you accept social work interns in your dental setting?

- No (1)
- Yes (2)

Display This Question:

If Do you accept social work interns in your dental setting? = Yes

Q18a What type of social work students do you accept? (Select all that apply)

- ☐ MSW (1)
- ☐ BSW (2)
- ☐ Other (3) ____

Display This Question:

If Do you accept social work interns in your dental setting? = Yes

Q18b In an average academic year, how many social work student interns do you host?

- ○ 1 (1)
- ○ 2 (2)
- ○ 3 (3)
- ○ 4 or more (4)

Display This Question:

If Do you accept social work interns in your dental setting? = Yes

Q18c Over the course of your position, what is the total number of social work student interns you have supervised? (Please include those who are currently placed with you)

____

End of Block: Social Work Questions

Start of Block: Financial Questions

FQ. The following section will ask you questions about how your program is funded.

Q19 How is the social work service/program at your dental school funded?

- ☐ Dental School Budget (1)
- ☐ Social Work School/Program Budget (2)
- ☐ Grant(s) (3)
- ☐ Clinical Revenue (5)
- ☐ Other Revenue Sources, please specify: (4) ____

Display This Question:

If How is the social work service/program at your dental school funded? = Dental School Budget

Or How is the social work service/program at your dental school funded? = Social Work School/Program Budget

Or How is the social work service/program at your dental school funded? = Grant(s)

Or How is the social work service/program at your dental school funded? = Other Revenue Sources, please specify:

Or How is the social work service/program at your dental school funded? = Clinical Revenue

Q19a What percent of your funding comes from the dental school, social work school, grant, and/or other? (Total should add to 100%)

	Dental School (1)	Social Work School/Program (2)	Grant(s) (3)	Clinical Revenue (4)	Other (5)
Percent (%) (1)					

Display This Question:

If How is the social work service/program at your dental school funded? = Grant(s)

Q19b What is the name of the grant(s) that supports funding for your service/office?

____

Q21 Do you bill insurance for your social work services?

- ○ No (1)
- ○ Yes (2)

Q21a What, if any, are barriers to billing for your social work services you have experienced?

____

End of Block: Financial Questions

Start of Block: Professional Development Questions

PDQ. The following section will ask you questions about your professional development and opportunities for professional development.

Q22 How do you secure professional development opportunities in your role?

____

Q23 What would be helpful professional development opportunities for you to have? (Please select all that apply)

- ☐ Clinical Hours (1)
- ☐ CEUs (2)
- ☐ In-service Trainings (3)
- ☐ Other, please specify: (4) ____

Q24 Do you have specific topics that you would like to have additional training on given your work/role?

____

End of Block: Professional Development Questions

Start of Block: Barriers & Facilitators

B&F. The following section will ask you questions about the barriers and facilitators that occurred in establishing your program.

Q25 Overall, what would identify as the major barriers to establishing social work services at your institution?

____

Q26 What have been the facilitators/factors that have helped establish social work services at your institution?

____

Q27 What else would like us to know about your social work role that was not included in this survey?

____

Q28 Would you be willing to be contacted for future research?

○ No (1)

○ Yes, my email is: (2) ____

End of Block: Barriers & Facilitators

8.2 Interview Guide

SWID SOCIAL WORKER INFORMATION:

SWID Member	Title	Email

INTRODUCTION

- **What the study is about:** The purpose of this research is to further investigate the emerging roles for social workers in dental education/clinic settings, the knowledge and skills required of social workers in these settings, and the barriers and facilitating factors to deploy social workers in dental education/clinic settings. Ultimately, we hope to expand the integration of social workers in dental education/clinical settings.
- **Do you have any questions?**
- **Recording:** If you agree, we will be audio recording this interview to better understand your responses and add to the analysis of the study. Your video will not be recorded. You have the option to keep your video on or off during the interview. **Is this okay?**

QUESTIONS

Content	Questions	Notes	Comments
Participant Introduction	• **Please tell me briefly about your role and some of the primary things you do at [institution name]?**<br>○ **How long have you been in your role?**	• This section of the questions is to get a brief introduction to the participant.	•

History of how they came to the role	• **How did you come to be a social worker in dentistry?**<br>o *What excited you about this opportunity?*<br>o *What did you think of when you came across/was introduced to this opportunity?*<br>o *What did your training look like for this role?*	• This section of questions is focused on how the participant came to this role.	•
Exploring the role of the participant in their institution	You told me about your role when we started, now I am going to ask you a few more specific questions about this.<br>• **What is your main role as a social worker in the dental school?**<br>o *How has the role changed throughout your time?*<br>o *What are other roles you have?*<br>• **What are some examples/activities of the different roles you play?**<br>• *How does your positionality impact your different roles?*	• This section aims to go in-depth on the participant's role in the dental school.	•
Exploring how others in the dental school perceive participants role in the dental school	• **Please describe a time/situation where you felt your social work services would have been beneficial but where not utilized?**<br>o *In what ways do you feel your team values and understands your role as a social worker?*<br>o *In what ways do you feel your team does not value or understand your*	• This section aims to understand what participant's feel others believe their role is and what the capacity of the role is.	•

	role as a social worker?		
Exploring participant's role in health equity	• **In your opinion, in what ways do social workers contribute to providing quality and equitable oral health care? Please give an example.**<br>○ *What do you believe has been your personal contribution?*<br>○ *In what ways have your contributions positively affected patients, dental students, and dental school administration?*	• There is a shared goal of health equity among dentists and social workers and so these questions is directly exploring perceptions and experiences of health equity in their role and as part of an interdisciplinary team.	•
Barriers and Facilitators of role	• **What are some of the challenges and/or barriers you face being a social worker in a dental education/clinic setting?**<br>• **What are some of the strengths and facilitators of being a social worker in a dental education/clinic setting?**<br>○ *What are some personal barriers/facilitators?*<br>○ *What are some institutional barriers/facilitators?*<br>○ *How have you overcome these challenges and/or barriers?*<br>○ *Do you feel as though you are part of an*	• This section of questions is to explore the barriers and facilitators of the role of the participant in the dental school.	•

	interprofessional team?<br>▪ *Can you provide examples why yes/no?*		
Wrap-up Question	• **Is there anything you would like to add about your experience and role that was not mentioned/asked?**	• This is the last question to be asked that wraps up the interview.	•

KEY:
BOLD – Main Questions to ask, *Italicized* – If we have time, ask these questions

MEMO

Questions	Facilitator Thoughts
Who is the participant and what is their background?	
How did the interview go?	
What main findings stand out, were surprising, or were new?	
